# CEOs' early-life disaster experience and corporate earnings quality: Focusing on the Great Chinese Famine

**DOI:** 10.3389/fpsyg.2022.1041630

**Published:** 2022-11-25

**Authors:** Yang Zhao, Jun Hu, Lang Liu

**Affiliations:** ^1^School of Management, Jinan University, Guangzhou, China; ^2^Management School, Hainan University, Haikou, China; ^3^Development and Planning Department, Jinan University, Guangzhou, China

**Keywords:** the Great Chinese Famine, risk prevention, learning effect, earnings quality, early-life disaster experience

## Abstract

This paper aims to examine the impact of CEOs' early-life disaster experiences on corporate earnings quality. We proxy the disaster experience with whether CEOs lived through the Great Chinese Famine and the famine intensity they experienced. The results indicate that CEOs' early-life famine experience is significantly positively associated with corporate earnings quality, and the famine effects are more obvious for CEOs who experienced the famine at adolescent ages. Further tests show that the famine experience effects are more pronounced in companies with high investor protection and cross-listing and with CEOs who have a relatively high level of education or background in economic management. The findings suggest CEOs would bear the imprint of an adverse early-life experience, which has risk aversion and learning effects on their decision making in corporate earnings information disclosure.

## Introduction

Imprinting theory holds that the process through which a focal entity develops characteristics that reflect prominent features of the environment, and these characteristics persist despite significant environmental changes (Kriauciunas and Kale, 2006; Marquis and Tilcsik, 2013). Based on this theory, a growing body of literature has shown that the early-life imprint on CEOs affects their management style (Bukalska, 2020; Górska and Mazurek, 2021), such as their professional background (Hitt and Tyler, 1991; Waller et al., 1995; Ding et al., 2021), military experience (Malmendier et al., 2011; Benmelech and Frydman, 2015), and financial experience (Frank and Goyal, 2003; Graham et al., 2013; Jiang et al., 2016). In this study, we regard CEOs' experience of the Great Chinese Famine as an imprint and investigate the impact of CEOs' early-life disaster experiences on corporate earnings quality.

Exploring the influencing factors of corporate earnings quality is one of the fundamental topics in accounting research. Corporate earnings information is the cornerstone of the efficient operation of the capital market, on which investors and information intermediaries' decision making are heavily dependent (Mao et al., 2013). The information of earnings quality directly affects the information asymmetry between the inside and outside of an enterprise, and to a large extent, it also affects the efficiency of the capital market (Kim and Verrecchia, 1994; Cheng et al., 2014; Jiang et al., 2016; Durana et al., 2021; Valaskova et al., 2021). Prior studies have shown that companies with high-quality earnings information can reduce external financing costs (Francis et al., 2005), alleviate financing constraints (Biddle and Hilary, 2006), and reduce litigation risk (Palmrose and Scholz, 2004) as well as bid-ask spreads (Handa et al., 2003). Therefore, it is a common concern and practice in academia in exploring the factors affecting corporate earnings quality.

Based on the imprinting theory, researchers have documented that CEOs' experiences of adversity can have a profound impact on their future managerial style. For example, Malmendier et al. (2011) found that CEOs who experienced the Great Depression are more cautious in debt financing and exhibit more biases toward internal financing. Bernile et al. (2017) found that CEOs who have experienced major disasters are more risk-averse, which results in companies with lower financial leverage, more cash holdings, and less mergers and acquisition (M&A) activities. Based on the Great Famine of 1959–1961 in China, studies found that the painful psychological imprint of this event has led people to have a higher desire for savings (Cheng and Zhang, 2011), pursue more conservative financial policies (Zhang, 2017; Feng and Johansson, 2018), and feel a greater sense of social responsibility (Xu and Ma, 2022). Although more research studies have been carried out on the long-term effects of CEOs' early-life experiences, there have been relatively few studies that focus on corporate earnings quality.

In addition, it is worth pointing out that although previous studies have found that CEOs' different early-life experiences have an important impact on corporate behavior, such as military experience (Malmendier et al., 2011; Benmelech and Frydman, 2015) and financial experience (Frank and Goyal, 2003; Graham et al., 2013), the results of these studies may induce selection bias problems. For example, an individual's personality is likely to play a role in their decision to join the army, which would confound the results regarding the effects of military experience. However, investigating differences in decision-making behaviors due to involuntary life experiences may effectively alleviate this problem. The Great Chinese Famine is a rare opportunity for us to explore the impact of CEOs' early-life experiences on their future corporate behaviors. First, it is difficult for most citizens to anticipate such a tragedy; however, it may be reasonable to assume the Great Chinese Famine to be a clear exogenous shock for CEOs living during that age. Moreover, although the famine affected almost all regions of China, people now over the age of 59 years have all experienced it (Chen and Yang, 2015); however, due to differences in natural resources, culture, and economic conditions in different regions, the impact of the famine was not the same everywhere in China (Cao, 2005). By identifying the intensity of the impact of the Great Famine in different places using the difference in differences (DID) method, we could alleviate the potential endogenous problems in estimating the influence of CEOs' early-life experience on corporate earnings quality.

To test our proposition, we manually collected CEOs' personal information (birthplace and age) to match it with the occurrence of the Great Famine and the severity of the famine's impact on different regions. We proxy the CEOs' famine experience with whether a CEO lived through the years of the Great Chinese Famine (1959–1961) and the famine's intensity in the CEOs' birthplace. Following prior studies (e.g., Lennox et al., 2016, 2018), we used the modified Jones model proposed by Dechow et al. (1995), which is widely used in the literature, to measure corporate earnings quality. Using the DID method and taking 2000–2015 Chinese listed companies as the sample, we found that CEOs' early-life famine experience is significantly positively associated with corporate earnings quality. Furthermore, by identifying the age at which CEOs experienced the famine, we showed that the effects of famine experience on corporate earnings quality are more pronounced for CEOs who had this experience at the adolescent stage. These results are consistent with the imprint theory and suggest that CEOs would bear an imprint of their adverse early-life experiences, which would influence their decision making in corporate earnings information disclosure.

We also examined two potential channels, namely, the risk aversion effect and the famine learning effect, to explain the relationship between CEOs' famine experience and corporate earnings quality. On the one hand, the risk aversion effect holds that the early-life famine experience makes CEOs more risk-averse toward earnings information disclosure as low-quality earnings may aggravate the risk of litigation. On the other hand, the famine learning effect holds that the epochal characteristics of and reasons for the occurrence of the Great Chinese Famine increase the knowledge of famine-experienced CEOs, which results in less earnings manipulation and high-quality earnings information. By dividing our sample into different groups, we found that the relationship between CEOs' Great Famine experience and earnings quality is more pronounced for firms with high investor protection and cross-listing and for CEOs with a relatively high level of education or a background in economic management. These results are consistent with the theories of risk aversion and famine learning effects, which suggest that the famine experience may influence CEOs' decision making through the two channels.

Our paper contributes to the literature in several ways. First, our paper deepens the understanding of the influences of corporate earnings quality. High-quality financial information is the cornerstone of the efficient operation of capital markets and has a significant impact on the efficient allocation of market resources and long-term enterprise development (Dechow et al., 2010). Prior studies have discussed the factors that may influence corporate earnings quality in relation to corporate governance structures (Dhaliwal et al., 2006), company growth (Collins and Kothari, 1989), equity concentration (Fan and Wong, 2002), information media (Bushee et al., 2010; Cheng et al., 2014), and policies and regulations (Cohen et al., 2008; Koh et al., 2008). However, taking CEOs' heterogeneity into account, this paper provides empirical evidence that the CEOs' early-life famine experience is significantly positively associated with corporate earnings quality, thereby further enriching and supplementing the relevant areas of literature on corporate earnings quality.

Second, our paper contributes to the literature related to CEOs' managerial styles using the imprint theory. A growing literature has explored the potential impacts of CEOs' managerial styles and their consequences, such as professional background (Waller et al., 1995), military experience (Malmendier et al., 2011; Benmelech and Frydman, 2015), and financial experience (Frank and Goyal, 2003; Graham et al., 2013). Our research indicates the significance of the impact of CEOs' experience of the Great Chinese Famine on corporate earnings quality using the imprint theory and provides two potential channels for explaining this relationship, enriching the literature on CEOs' managerial style. Further, due to the exogenous and unpredictable nature of the Great Famine, our paper also provides a more robust estimate of the impact of CEOs' early-life experiences on corporate decision making, thereby addressing the selection bias in the existing literature that considered financial experience, overseas experience, and military experience of CEOs.

Third, concerning corporate earnings quality, our paper also enriches the literature related to the long-term economic consequences of the Great Chinese Famine. Prior studies have documented several impacts and outcomes of the Great Famine, but most focused on social psychology (Chen and Zhou, 2007; Meng and Qian, 2009), macroeconomics (Harbaugh, 2004), and corporate finance (Zhang, 2017; Feng and Johansson, 2018; Hu et al., 2019, 2020). Complementing the existing literature, this paper examined the event's impact on corporate earnings quality and found that CEOs' early-life famine experience significantly positively affected the quality of corporate earnings. It was also found that this effect was more pronounced for CEOs who experienced the famine at the adolescent age; this finding enriches the literature on the long-term economic consequences of the Great Chinese Famine.

The remainder of this paper is organized in the following. The second section presents the literature review and research hypotheses. Materials and methods section describes the research design. The fourth section reports the empirical results. The fifth section presents the robustness tests. The sixth section discusses the results of further analyses. The seventh section provides the discussion of this study. The final section provides the limitation and future research.

## Literature review and research hypotheses

### The 1959–1961 Great Famine in China

**Figure 1:**
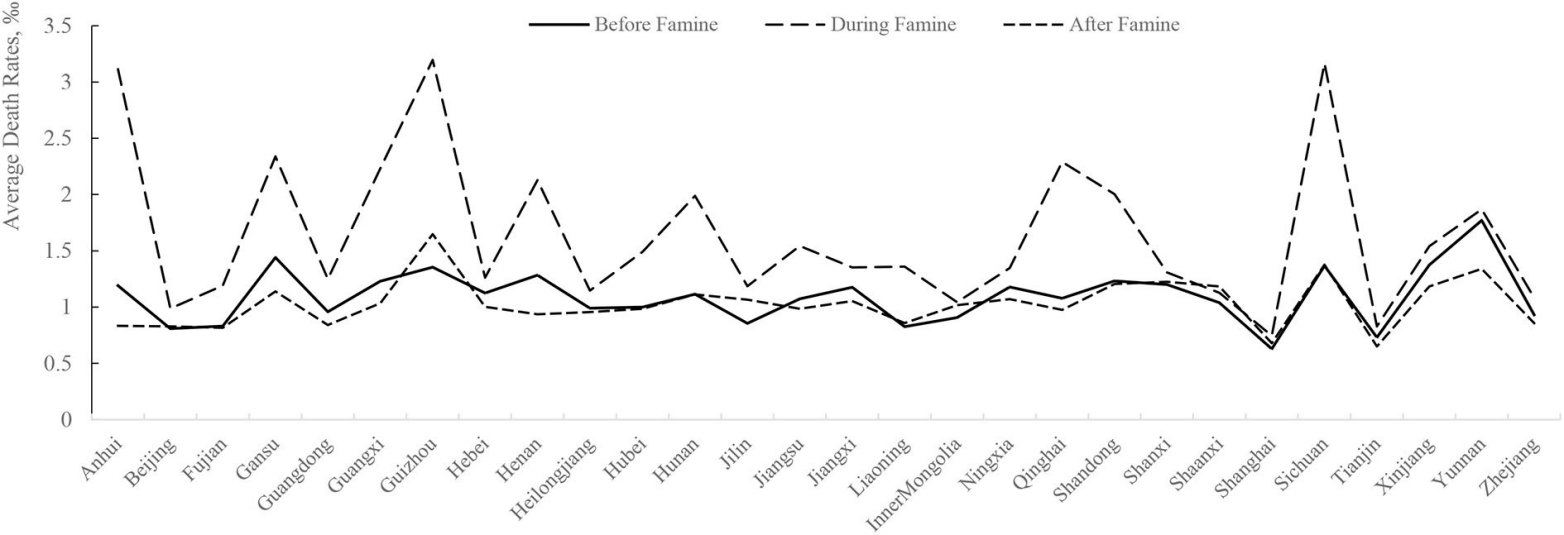
Distribution of the severity of the famine by province. This figure shows the distribution of the severity of the famine by province. The sample is from 1956 to 1964. The X-axis is 28 provinces in China. Tibet is not listed because of lacking data. The Y-axis is the average mortality rate for each province. We calculate the average mortality rate in three ways: 3 years before the *Great Famine*, 3 years during the *Great Famine*, and 3 years after the *Great Famine*.

China is one of the most famine-affected countries in the world and has even been referred to as “a land of famine” by scholars (Mallory, 1926). Historically, the Great Chinese Famine of 1959–1961 was the most recent famine in China. It had a significant impact on the population, economy, and social development at that time. Figure 1 presents the average mortality rates in every province over the 3 years before, during, and after the famine. From the figure, we can infer the following: first, this famine had a wide impact. During the famine, the average mortality rate of all provinces in China showed a significant upward trend, indicating that almost all provinces and regions were affected (Chen and Yang, 2015). Second, the impact was different in different regions. For example, during the famine, the average mortality rate in Anhui, Guizhou, and Sichuan provinces exceeded 3%, which was significantly higher than that in other provinces. Third, the impact faded rapidly, as the average mortality rate of 3 years after the famine returned to the rate of 3 years before it.

### Consequences of the Great Chinese Famine

The Great Chinese Famine of 1959–1961 was one of several extraordinary natural disasters in China for which more data are available. It had a profound impact on both the social and economic development of the region. Related topics have been discussed by researchers in the fields of sociology, economics, and finance. Sociological researchers focused on the long-term effects of the famine on personal growth and found that the starvation and abnormal deaths during the Great Chinese Famine had an irreversible impact on the future of survivors, resulting in smaller populations, lower body weights, and reduced labor force supply (Chen and Zhou, 2007; Meng and Qian, 2009). Further, the health status of the generation who experienced the famine was also relatively poor (Ma, 2011).

Studies on the long-term effects of the Great Chinese Famine on macroeconomic and individual behaviors have accumulated in the literature, providing rich evidence. For example, Gooch (2017) found that although the state of the country returned to how it was 3 years before the famine within 3 years after it, this event had a significant negative impact on the economic growth of the country in the long run. Moreover, due to the memory of the famine, individuals who experienced the early-life famine tend to pay closer attention to food storage (Cao, 2005), exhibiting a greater tendency to save (Harbaugh, 2004). Wang et al. (2015) found that experiencing the famine early in life had a major impact on individuals' self-employment choices: it made such individuals more risk-averse, leading them to avoid starting up a business.

Finance scholars have also examined the impact of the Great Famine experience on corporate behavior. O'Sullivan et al. (2021) found that an experience of trauma early in a CEO's life is positively associated with corporate social performance. Xu and Ma (2022) found that firms with CEOs who experienced early-life poverty are associated with more socially responsible activities and fewer socially irresponsible activities. Zhang (2017) showed that CEOs who experienced the famine had a higher risk aversion tendency, which manifested itself in lower debt levels, more cash holdings, and lower probability of engaging in M&A activities. Zhou et al. (2022) demonstrated that CEOs' early-life adversity experiences have a significantly positive effect on firm internationalization. Concerning accounting and finance policies, Feng and Johansson (2018) found that companies with CEOs having lived through the famine are associated with more conservative financial, investment, and cash-holding policies, a lower likelihood of unethical behavior, and better firm performance during economic downturns. Hu et al. (2019) found that CEOs who experienced the Great Famine (1959–1961) hold higher amounts of cash and increased cash sensitivity. Hu et al. (2020) found that accounting conservatism was significantly higher for companies whose CEOs have experienced the Great Chinese Famine. Yao et al. (2020) found firms led by top executives who experienced the Great Famine in early life are less likely to conduct fraudulent financial reporting. Long et al. (2020) found that companies whose CEOs experienced the “Great Chinese Famine” in early life have lower stock price crash risk than those with CEOs who did not experience the famine. Using the Korean War as an exogenous shock, Choi and Jung (2021) found that firms managed by war-exposed CEOs have greater information transparency than firms managed by CEOs who are not war-exposed.

### Hypotheses development

From existing studies, we know that the Great Chinese Famine left an indelible imprint on the psychological growth of individuals who experienced the disaster, which would affect individuals' future behavioral decisions (Zhang, 2017; Hu et al., 2019). Drawing on the related studies, we predict that CEOs' famine experience has the following two effects on corporate earnings quality.

On the one hand, the painful memory of the Great Chinese Famine makes a CEO more risk-averse, which could improve corporate earnings quality. This is the “risk aversion effect.” The existing literature found that early disaster experience has a long-term physical and psychological impact on CEOs, leading to a higher degree of risk aversion. This could affect corporate earnings quality in various ways. Corporate earnings information is the cornerstone of the efficient operation of capital markets, on which investors and information intermediaries' decision making are heavily dependent (Mao et al., 2013). For corporate CEOs, although low-quality earnings information disclosure can increase investor information asymmetry, which may be beneficial for managers' opportunistic behavior and obtaining excess returns, it may also lead to a higher uncertainty and litigation risk (Field et al., 2005). According to the imprint theory, individuals are imprinted by adverse early-life experiences (Marquis and Tilcsik, 2013), famine-experienced CEOs are more risk-averse and precautionary about uncertainties (Zhang, 2017; Hu et al., 2019), and thus, they are more likely to overstate the possible risk and understate the benefit of earnings management, which would reduce the incentive to earnings management and result in a high quality of earnings disclosure.

On the other hand, the background and causes of the famine may increase CEOs' knowledge and cognition, and the famine learning effect may also improve corporate earnings quality. Previous literature has found that the *catch-up strategy* employed during the Great Leap Forward and the associated over-purchase of food were important causes of abnormal deaths during the Great Famine (Kung and Lin, 2003). Psychological research has shown that individual decision making is rooted in the memory of past experiences and conditioned reflexive learning, which form a sort of knowledge guide for current actions (Freud, 1915; Schlag, 1998). The famine learning effect may be recognized in CEOs' reflection on the negative consequences of the exaggerated behavior (e.g., catch-up strategy) during the Great Leap Forward, which ultimately affects their motivation to manipulate corporate earnings. Feng and Johansson (2018) found that CEOs' early-life famine experience increased corporate conservatism, and such CEOs rarely cheated. CEOs who are influenced by the famine learning effect can be expected to pursue less earnings manipulation and have relatively higher requirements for the quality of their corporate information disclosures, which may improve corporate earnings quality.

Therefore, the risk prevention effect and the famine learning effect produced by the famine experience may lead the CEO to have higher requirements for corporate earnings quality. Accordingly, we formulate our first hypothesis, which is presented as follows:

*H1: Controlling for other factors, CEOs' famine experience is positively associated with corporate earnings quality*.

Differences in cognitive ability can be found between different age groups. Psychological research indicates that adolescence is the most crucial phase in the formation of individual thinking and values. During this period, experiences with clothing, food, cold, and warmth play a key role in the formation of individual qualities, psychological tendencies, and personality structure (Elder Jr et al., 1991; Becker, 1992). Since adolescence is the key stage in the formation of cognitive abilities and personality traits, the imprint left by the Great Chinese Famine at this stage may have a larger and more profound impact on personal behavior. By distinguishing between the birth cohorts of family heads, Cheng and Zhang (2011) found that famine experience during adolescence has a greater impact on an individual's propensity to save. Feng and Johansson (2018) showed that famine experience during CEOs' childhood or adolescence has a more pronounced impact on their corporate decision making than famine experience in other life stages. Based on the above studies, we expect that CEOs who experienced the Great Chinese Famine during adolescence would have a more pronounced effect on corporate earnings quality than those who experienced it in other ages. We propose the second hypothesis as follows:

*H2: The experience of the Great Famine during adolescence has a more significant positive effect on corporate earnings quality than that at another life stage*.

## Materials and methods

### Data and sample

Our original sample includes all companies listed on the Chinese A-share market, and we obtained financial data from the China Stock Market and Accounting Research (CSMAR) database. The CSMAR database contains financial and corporate governance information from 1999 to date. However, our sample was limited to 2000 to 2015 because observations for some test variables in 1999 are missing from the database. Our sample ending range is 2015 because the CEOs' background information that we manually collected has been limited to 2015.

We applied the following exclusion criteria: samples from the financial and insurance industry; ST and ^*^ST listed companies; samples whose CEOs were of a non-Chinese nationality or born in Taiwan, Hong Kong, Macau, or Tibet; and samples with missing financial information or missing place of CEO's birth. To control the influence of outliers, all continuous variables were winsorized at the 1 and 99% quantiles. Ultimately, we obtained 23,974 enterprise-year samples.

### Variables

#### Earnings quality

We used the modified Jones model proposed by Dechow et al. (1995) to measure earnings quality. The following calculation method was used. First, we used a cross-section regression estimation model (1) to obtain the parameters k_1_, k_2_, and k_3_.


(1) $$\begin{array}{l} \frac{TACC_{t}}{TA_{t-1}}=k_{1}\frac{1}{TA_{t-1}}+k_{2}\frac{\text{Δ}Sales_{t}}{TA_{t-1}}+k_{3}\frac{\text{Δ}PPE_{t}}{TA_{t-1}}+\varepsilon \end{array}$$

In model (1), *TACC*_*t*_ represents the total accrued profits in year t, calculated by *NI*_*t*_-*CFO*_*t*_, where *NI*_*t*_ represents the net profit in year t and *CFO*_*t*_ represents the net cash flow from operating activities in year t; *TA*_*t*−1_ is the total assets in year t−1; Δ*Sales*_*t*_ represents the changes in corporate operating income calculated by the difference between corporate operating income in years t and t−1; and *PPE*_*t*_ represents the net amount of fixed assets in year t.

Second, we calculated the controllable accruals of the sample companies based on model (2), as follows:


(2) $$\begin{array}{l} DACC_{t}=\frac{TACC_{t}}{TA_{t-1}}-({\hat{k}}_{1}\frac{1}{TA_{t-1}}+{\hat{k}}_{2}\frac{\text{Δ}Sales_{t}-\text{Δ}AR_{t}}{TA_{t-1}}+\\ \;{\hat{k}}_{3}\frac{PPE_{t}}{TA_{t-1}})\; \end{array}$$

*DACC*_*t*_ represents discretionary accruals in year t; Δ*AR*_*t*_ is the change in corporate receivables; and the estimated values for parameters k_1_, k_2_, and k_3_ are estimated from model (1). Specifically, *DACC*_*t*_ is the degree to which actual discretionary accruals of an enterprise deviate from ideal discretionary accruals. The greater the deviation, the more severe the earnings management. We take the absolute value of *DACC*_*t*_ obtained by model (2) and expressed it as *EQ*_*t*_. The larger the *EQ*_*t*_, the worse the earnings quality, and the smaller the *EQ*_*t*_, the better the earnings quality.

#### CEO famine experience

The Great Chinese Famine had widespread and far-reaching effects, but as shown in Figure 1, its severity was different in different regions. Referring to Chen and Zhou (2007), Cheng and Zhang (2011), and Wang et al. (2015), we used CEOs' birth year and birthplace to identify who had experienced the Great Famine. First, we relied on CEOs' birth year to define whether a CEO was living through the Great Famine. If a CEO was born in 1961 or before, we defined such CEOs as famine-experienced CEOs and encoded *famine experience* to 1 and otherwise encoded to 0. Second, we performed the following steps to measure famine intensity: calculate the difference in average death rates between 1959–1961 and 1956–1958 for each province and the difference in average death rates between 1959–1961 and 1962–1964 for each province; estimate the mean average excess death rate (*EDR*) using these two differences to obtain the *EDR* for each province during 1959–1961. Further, based on the *EDR*, we set a dummy variable *EDD*, which was encoded to 1 if the province experienced a median-above *EDR* during the three famine years and otherwise 0. Finally, following Hu et al. (2020) and Long et al. (2020) we constructed two interaction variables to capture CEOs' famine experience: Famine^*^EDRandFamine^*^EDD. It can exclude the impact of CEOs' age on the conclusion.

#### Control variables

Following Lennox et al. (2016), we selected control variables associated with corporate earnings quality. *Size* is the natural logarithm of the company's total assets at the end of the year t. *Lev* is the company's total liabilities scaled by total assets at the end of the year t. *ROA* is the return on assets, calculated by the net profit divided by total assets at the end of the year t. *Grow* is sales revenue growth, defined by the annual sales revenue minus the previous year's sales revenue and then scaled by the previous year's sales revenue. *SOE* is a dummy variable, defined as 1 if a company is state-owned and otherwise 0. *Audit* is a dummy variable that is encoded to 1 if the company is audited by a Big 4 or 5 audit firm and otherwise 0. *Dual* is 1 if the CEO is also the chairman of the company and otherwise 0. *OCF* is the free cash flow, which is the net cash flow of the company scaled by total assets at the end the year t. First is the ratio of shares held by the largest shareholder of the company. *IO* is the share ratio for institutional investors. Regarding managerial characteristics, *Age* is the natural logarithm of CEOs' age; *Gender* is a dummy variable that is encoded to 1 if the CEO is female and 0 otherwise; *Degree* is the natural logarithm of 1 plus CEOs' degree, which is assigned the value of 1, 2, 3, 4 or 5 if the CEO has a middle school, high school, college, master, or doctoral degree, respectively. Detailed definitions of all these variables are given in Appendix 1.

### Model

It should be noted that age is an important factor for managers' risk preference and corporate earnings quality. For example, older managers are more inclined to avoid risk than younger ones (Yim, 2013; Serfling, 2014), and as such, the reported earnings quality of their corporations tends to be higher (Huang et al., 2012). Since CEOs who experienced the Great Chinese Famine tend to be older, age may be an alternative explanation for our hypothesis. To address this issue, we set up a treatment group and a control group, which were distinguished by whether the CEO experienced the famine and the degree of famine intensity in the region where the CEO was born. We used a DID method to test the effect of CEOs' famine experience on corporate earnings quality. The model is as follows:


(3) $$\begin{array}{l} EQ_{i,t}=\beta_{0}+\beta_{1}Famine_{i,t}+\beta_{\text{2}}Famine_{i,t}\text{*}EDR_{i,t}(EDD_{i,t})+\\ \;\beta_{\text{3}}EDR_{i,t}(EDD_{i,t})\;\\ \;+Ctrls+Y+I+P+BP+BY+\varepsilon \end{array}$$

In model (3), *EQ* indicates corporate earnings quality; famine experience is the independent variable that is proxied by Famine^*^EDR (*EDD*); *Ctrls* are the control variables as described above. Further, due to the economic cycle, industry environment, regional factors, and CEO growth environment may affect the validity of the regression estimation. Thus, in order to control the different fixed effects, we added five dummy variables to our model: year (Y), industry (I), province (P), CEO birthplace (BP), and CEO birth year (BY).

It is worth noting that although the famine experience is an interaction term for *Famine* and *EDR* (*EDD*), these two variables do not change with time and province. While we controlled the fixed effects of BY and BP, we did not add the variables *Famine* and *EDR* (*EDD*) to the model again. In model (3), our variable of interest is Famine^*^EDR (EDD). If our hypothesis is true, we can expect that the coefficient of β_2_ will be negative and significant even for CEOs who experienced the famine during the same age as different intensities of famine in different regions may lead to differences in earnings quality.

## Empirical results

### Descriptive statistics

Table 1 reports the descriptive statistics of the main variables in this paper. The average earnings quality (*EQ*) is 0.047, and the median is 0.036, but the standard deviation is 0.051, indicating a large difference in earnings quality in the sample. The average value of *Famine* is 0.419, indicating that about 42% of the CEOs in the sample experienced the Great Chinese Famine. The average *EDR* is 0.591%, which indicates that the average excess mortality rate during the 3-year famine is close to 0.6%. Given China's large population, this number indicates a painful and profound impact. The standard deviation of the *EDR* is 0.534, which is close to the average, indicating that the impact of the famine differed greatly in different provinces. The descriptive statistics of other control variables are mostly consistent with prior studies and have not been described in detail in this paper.

**Table 1:** Descriptive statistics of the main variables.

**Variable**	***N***	**Mean**	**Std dev**.	**Q1**	**Median**	**Q3**
EQ	23,974	0.047	0.051	0.016	0.036	0.055
Famine	23,974	0.419	0.493	0.000	0.000	1.000
EDR (%)	23,974	0.591	0.534	0.190	0.368	0.785
EDD	23,974	0.498	0.500	0.000	0.000	1.000
Size	23,974	21.650	1.231	20.805	21.503	22.313
Lev	23,974	0.474	0.230	0.310	0.472	0.623
ROA	23,974	0.032	0.065	0.012	0.033	0.061
Grow	23,974	0.163	0.348	0.000	0.093	0.224
SOE	23,974	0.575	0.494	0.000	1.000	1.000
Audit	23,974	0.069	0.254	0.000	0.000	0.000
Dual	23,974	0.176	0.380	0.000	0.000	0.000
OCF	23,974	0.045	0.078	0.003	0.044	0.089
First	23,974	0.377	0.157	0.252	0.362	0.495
IO	23,974	0.168	0.200	0.012	0.085	0.258
Age	23,974	3.849	0.141	3.761	3.850	3.951
Gender	23,974	0.052	0.221	0.000	0.000	0.000
Degree	23,974	1.431	0.214	1.386	1.386	1.609

This table presents the sample descriptive statistics. EQ is the corporate earnings quality, calculated by the modified Jones model. Famine was encoded to 1 if the CEO was born in 1961 or earlier and 0 otherwise. EDR is the excess death ratio, valued as the mean difference of mortality in the given province during 3 years of famine subtracted from the average mortality before and after 3 years of the Great Famine. EDD is a dummy variable, which is encoded to 1 if the provinces have a median-above EDR during 3 years of famine and otherwise encoded to 0. Size is the natural logarithm of the total assets of the company at the end of the year. Lev is the total liabilities of the company scaled by total assets at the end of the year. ROA is the return on assets, defined by the net profit divided by total assets at the end of the year. Grow is sales revenue growth, defined by the annual sales revenue minus the previous year's sales revenue and then scaled by the previous year's sales revenue. SOE is a dummy variable, defined as 1 if a company is state-owned and otherwise defined as 0. Audit is a dummy variable that if the company audited by a Big 4 or 5 audit firm is encoded to 1 and 0 otherwise. Dual is 1 if the CEO is also the company's chair and 0 otherwise. OCF is the free cash flow, using the company's net cash flow scaled by total assets at the end the year. First is the shares ratio held by the largest shareholder. IO is the share ratio for institutional investors. Age is the natural logarithm of CEO age. Gender is a dummy variable which is encoded to 1 if the CEO is a female and 0 otherwise. Degree is the natural logarithm of 1 plus CEOs' degree, which is assigned 1, 2, 3, 4, or 5 if CEO has the middle school, high school, college, master's, or doctoral degree, respectively. Appendix 1 provides a detailed description of all variables.

Based on the intensity of the famine in the region where the CEO was born, we further described the association between CEOs' famine experience and the distribution of the earnings quality. The results are given in Table 2. In the table, the first column shows the distribution of CEO birth areas in our sample, and the second column shows the population *EDR* in each region from 1959 to 1961. As shown in the table, *EQ* is relatively higher among CEOs who were born in provinces with higher mortality, indicating the average earnings quality is slightly lower for CEO who did not experience the famine.

**Table 2:** Descriptive statistics of CEO birthplace distribution, excess death rate (EDR) for each province during the famine period and corporate earnings quality.

**CEO birthplace distribution**	**Excess death rate (EDR)**	**Famine CEOs, EQ**		**Non-Famine CEOs, EQ**	
		**CEOs**	**Mean EQ**	**CEOs**	**Mean EQ**
Anhui	2.102	351	0.037	573	0.043
Beijing	0.169	420	0.042	508	0.053
Chongqing	1.796	116	0.039	218	0.049
Fujian	0.368	303	0.046	497	0.046
Gansu	1.049	117	0.032	215	0.050
Guangdong	0.350	880	0.050	1,253	0.051
Guangxi	1.098	122	0.035	137	0.047
Guizhou	1.695	90	0.035	111	0.049
Hainan	0.350	129	0.061	65	0.052
Hebei	0.197	385	0.053	371	0.046
Henan	1.019	405	0.037	627	0.046
Heilongjiang	0.174	212	0.048	248	0.051
Hubei	0.500	465	0.041	755	0.054
Hunan	0.875	539	0.039	1,051	0.046
Jilin	0.225	279	0.045	287	0.049
Jiangsu	0.515	863	0.038	1,165	0.048
Jiangxi	0.238	262	0.045	402	0.045
Liaoning	0.517	463	0.039	471	0.052
Inner Mongolia	0.082	104	0.044	150	0.045
Ningxia	0.106	40	0.043	60	0.043
Qinghai	1.263	30	0.043	39	0.039
Shandong	0.785	780	0.038	1,127	0.050
Shanxi	0.096	244	0.047	264	0.051
Shaanxi	0.015	230	0.048	456	0.056
Shanghai	0.093	679	0.049	457	0.060
Sichuan	1.796	440	0.039	627	0.050
Tianjin	0.137	137	0.051	198	0.049
Xinjiang	0.263	106	0.051	101	0.040
Yunnan	0.313	83	0.051	211	0.042
Zhejiang	0.190	766	0.044	1,290	0.048

This table presents the descriptive statistics of CEO birthplace distribution, excess death rate (EDR) for each province during the famine period, and corporate earnings quality. EQ is the corporate earnings quality, calculated by the modified Jones model. Famine CEOs was encoded to 1 if the CEO was born in 1961 or earlier and 0 otherwise. EDR is the excess death ratio, valued as the mean difference of mortality in the given province during 3 years of famine subtracted from the average mortality before and after 3 years of the Great Famine. Appendix 1 provides a detailed description of all the variables.

**Figure 2:**
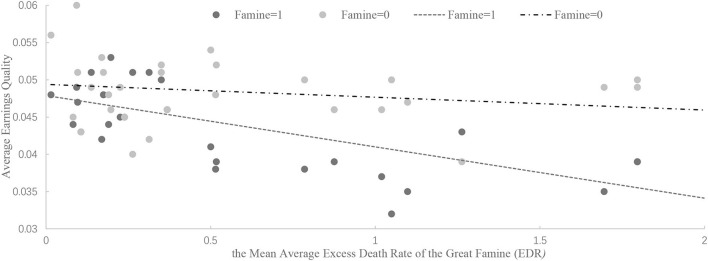
Trend graph of the mean average excess death rate of the *Great Famine* (EDR) and the average corporate earnings quality. This figure shows the mean average excess death rate of the *Great Famine* (EDR) and the average corporate earnings quality. The sample is from Table 2. The X-axis is the mean average excess death rate (EDR). We use the following methods to calculate the EDR: calculating the difference in the average death rate between 1959–1961 and 1956–1958 for each province, and the difference in average death rates between 1959–1961 and 1962–1964 for each provinces; and then calculating the mean average excess death rate (EDR) using the above two differences to obtain the EDR for each province during 1959–1961. The Y-axis is the average earnings quality for each province. We draw two lines based on whether the CEO experienced the *Great Famine*.

To intuitively describe the relationship between famine experience and earnings quality, we plotted the excess mortality rate in a CEO's birth place during the famine and the company's earnings quality based on descriptive statistical analyses. As shown in Figure 2, the straight line represented by *Famine* = 1 is lower than the straight line of *Famine* = 0, indicating that famine experience improves corporate earnings quality. Regardless of *Famine* = 1 or 0, corporate earnings quality increases with an increase in the impact of the famine (the trend line slopes downward to the right). Compared with *Famine* = 0 companies, the downward trend represented by *Famine* = 1 companies is more obvious. These results suggest that when a CEO experiences Great Famine, their company's earnings quality improved significantly, and the more severe the famine, if experienced, the higher the earnings quality.

### Univariate test

Before regression analyses, we performed a univariate test on earnings quality to check whether CEOs experienced the famine and the extent of famine intensity in CEOs' birthplace (*EDR*). The results are given in Table 3. For CEOs who did not experience the Great Famine (*Famine* = 0), no significant difference was seen in the mean and median of *EQ* between the *EDD* = 0 and *EDD* = 1 groups. However, for CEOs who experienced the famine (*Famine* = 1), the average *EQ* and median *EQ* in the *EDD* = 1 group were 0.038 and 0.036, respectively, which were significantly lower than those in the *EDD* = 0 group. This indicates that CEOs' famine experience significantly improved earnings quality. We also tested the DID results for *EQ* in the famine and *EDD* groups. The difference of the mean and median *EQ* was −0.008 and −0.002, respectively, both of which are significant at the 10% level at least. These results indicate that whether a CEO experienced the famine and the extent to which he experienced it have a significant impact on earnings quality.

**Table 3:** Univariate analyses, grouped by whether the CEO experienced famine and the severity of the famine.

**Variable = EQ**	**Famine** = **0**		**Famine** = **1**		**Differences**	
	**Mean**	**Median**	**Mean**	**Median**	**Mean**	**Median**
*EDD* = 0	0.050	0.035	0.048	0.039	−0.002^**^	0.004^***^
*EDD* = 1	0.049	0.034	0.038	0.036	−0.010^***^	0.002^***^
Differences	−0.001	−0.001	−0.009^***^	−0.003^***^	−0.008^***^	−0.002^*^

This table presents univariate analyses on earnings quality based on whether the CEO experienced the famine and the extent of the famine suffered in the CEO's birth region (EDD). EQ is the corporate earnings quality, calculated by the modified Jones model. Famine was encoded to 1 if the CEO was born in 1961 or earlier and 0 otherwise. EDD is a dummy variable which is encoded to 1 if the provinces have a median-above EDR during 3 years of famine and otherwise encoded to 0.

^***^, ^**^, and ^*^ represent statistical significance at the 1%, 5%, and 10% levels, respectively, based on p-values from t-tests. See Table 1 for variable definitions.

### Baseline regression

Table 4 reports the baseline regression results for the impact of CEOs' famine experience on corporate earnings quality. In columns (1) and (3), we did not include the control variables but controlled the fixed effect of industry, year, province, CEO birthplace, and CEO birth year. The coefficient of the interaction term Famine^*^EDR is −0.352 and that of the interaction term Famine^*^EDD is −0.007; both are significant at least at the 5% level. In columns (2) and (4), all firm-level and CEO-level control variables were controlled in the regresses. The coefficient of Famine^*^EDR was significantly negative at the 5% level in column (2), and Famine^*^EDD in column (4) was significantly negative at the 1% level. It should be noted that since the fixed effect of the birthplace and birth year of the CEO was considered, the influence of famine experience expressed by *Famine* and the intensity of famine expressed by *EDR* (*EDD*) were absorbed by the fixed effect of birth year and birthplace, and their coefficients were omitted due to collinearity (similar to subsequent regressions). Overall, the regression results given in Table 4 show that CEOs' famine experience significantly improved earnings quality of the company, which is in line with our basic expectations and supports H1.

**Table 4:** Regression results of CEOs' famine experience and corporate earnings quality.

	**(1)**	**(2)**	**(3)**	**(4)**
	**EQ**	**EQ**	**EQ**	**EQ**
Famine^*^EDR	−0.352^**^	−0.272^**^		
	(−2.51)	(−2.00)		
Famine^*^EDD			−0.007^***^	−0.006^***^
			(−4.17)	(−3.66)
Size		−0.005^***^		−0.005^***^
		(−7.29)		(−7.25)
Lev		0.022^***^		0.022^***^
		(6.54)		(6.52)
ROA		−0.016^*^		−0.016^*^
		(−1.76)		(−1.76)
Grow		0.016^***^		0.016^***^
		(9.47)		(9.46)
SOE		−0.002^***^		−0.002^***^
		(−2.58)		(−2.60)
Audit		−0.003^*^		−0.003^*^
		(−1.83)		(−1.75)
Dual		−0.000		−0.000
		(−0.11)		(−0.04)
OCF		0.008		0.008
		(1.44)		(1.45)
First		0.007^***^		0.007^***^
		(2.82)		(2.86)
IO		0.002		0.002
		(1.09)		(1.11)
Age		0.058^**^		0.058^**^
		(2.03)		(2.03)
Gender		0.002		0.002
		(1.24)		(1.28)
Degree		−0.007^***^		−0.006^***^
		(−2.88)		(−2.82)
Year	Yes	Yes	Yes	Yes
Industry	Yes	Yes	Yes	Yes
Province	Yes	Yes	Yes	Yes
Birth place	Yes	Yes	Yes	Yes
Birth year	Yes	Yes	Yes	Yes
*N*	23,974	23,974	23,974	23,974
Adj-R^2^	0.090	0.113	0.091	0.114

This table reports the regression results for famine experience on earnings quality. EQ is the corporate earnings quality, calculated by the modified Jones model. Famine was encoded to 1 if the CEO was born in 1961 or earlier and 0 otherwise. EDR is excess death ratio, valued as the mean difference of mortality in the given province during 3 years of famine subtracted from the average mortality before and after 3 years of the Great Famine. EDD is a dummy variable which is encoded to 1 if the provinces have a median-above EDR during 3 years of famine and otherwise encoded to 0. Size, Lev, ROA, Grow, SOE, Audit, Dual, OCF, First, IO, Age, Gender, and Degree are control variables. Appendix 1 provides a detailed description of all the variables. The t-value is given within parentheses based on robust standard errors clustered by the firm level.

^***^, ^**^, and ^*^ represent statistical significance at the 1%, 5%, and 10% levels, respectively.

The control variables for the size of the company (*Size*), the return on assets (*ROA*), the nature of property rights (*SOE*), audited by one of the Big 4 or 5 audit firms (*Audit*), and CEOs' education degree (*Degree*) were significantly negatively correlated with *EQ*, indicating that when an enterprise is larger, its return on assets is higher, it is state-owned, it is audited by a major accounting firm, or the CEO has a higher degree, the quality of corporate earnings is relatively higher. Financial leverage (*Lev*), company growth (*Grow*), the largest shareholder's ratio of shares (*First*), and the age of a CEO are significantly positively related to *EQ*, indicating that when companies have a higher debt ratio, they experience a higher speed of revenue growth and have more shareholding by the largest shareholder; likewise, when the CEO is relatively old, the earnings quality is worse. The results for the control variables are basically consistent with the findings of previous research (Fan and Wong, 2002; Lennox et al., 2016).

### CEO birth cohort effect

According to H2, the period of adolescence is a key stage for individuals' formation of cognitive abilities and personality traits, so the influence of famine on a CEO at this stage is more profound, and as such, the impact on earnings quality is more obvious. To test this hypothesis, we referred to Cheng and Zhang (2011) and further divided the age at which famine was experienced into infancy (<3 years), childhood (3–6 years), adolescence (7–17 years), and adult years (18 years and older) and named as *Cohort*_1_, *Cohort*_2_, *Cohort*_3_, and *Cohort*_4_, respectively. The regression results given in Table 5 show that the famine experience of CEOs of the difference cohorts has different effects on corporate earnings quality.

**Table 5:** Regression results for CEO age during the famine and the corporate earnings quality.

	**(1)**	**(2)**
	**EQ**	**EQ**
${\text{Cohort}}_{1}^{*}$EDR	−0.028	
	(−0.15)	
${\text{Cohort}}_{2}^{*}$EDR	−0.212	
	(−1.55)	
${\text{Cohort}}_{3}^{*}$EDR	−0.590^***^	
	(−2.90)	
${\text{Cohort}}_{4}^{*}$EDR	0.417	
	(1.43)	
${\text{Cohort}}_{1}^{*}$EDD		−0.002
		(−1.11)
${\text{Cohort}}_{2}^{*}$EDD		−0.004^**^
		(−2.03)
${\text{Cohort}}_{3}^{*}$EDD		−0.009^***^
		(−4.37)
${\text{Cohort}}_{4}^{*}$EDD		0.002
		(0.59)
Controls	Yes	Yes
Year	Yes	Yes
Industry	Yes	Yes
Province	Yes	Yes
Birth place	Yes	Yes
Birth year	Yes	Yes
*N*	23,974	23,974
Adj-R^2^	0.114	0.114
${\text{Cohort}}_{3}^{*}$EDR(EDD) = ${\text{Cohort}}_{1}^{*}$EDR(EDD)	*F* = 5.47^**^, *P* > *F* = 0.019	*F* = 3.54^*^, *P* > *F* = 0.063
${\text{Cohort}}_{3}^{*}$EDR(EDD) = ${\text{Cohort}}_{2}^{*}$EDR(EDD)	*F* = 3.46^*^, *P* > *F* = 0.063	*F* = 3.24^*^, *P* > *F* = 0.072
${\text{Cohort}}_{3}^{*}$EDR(EDD) = ${\text{Cohort}}_{4}^{*}$EDR(EDD)	*F* = 8.88^***^, *P* > *F* = 0.002	*F* = 10.55^***^, *P* > *F* = 0.001

This table reports the regression results for CEO age during the famine and corporate earnings quality. EQ is the corporate earnings quality, calculated by the modified Jones model. Cohort_1_, Cohort_2_, Cohort_3_, and Cohort_4_ were encoded to 1 if the CEOs experienced the famine in their infancy (<3 years old), childhood (3–6 years), adolescence (7–17 years), and adulthood (18 years and older), respectively and 0 otherwise. EDR is the excess death ratio, valued as the mean difference of mortality in the given province during 3 years of famine subtracted from the average mortality before and after 3 years of the Great Famine. EDD is a dummy variable which is encoded to 1 if the provinces have a median-above EDR during 3 years of famine and otherwise encoded to 0. Controls include Size, Lev, ROA, Grow, SOE, Audit, Dual, OCF, First, IO, Age, Gender, and Degree. Appendix 1 provides a detailed description of all the variables. The t-value is given within parentheses based on robust standard errors clustered by the firm level.

^***^, ^**^, and ^*^ represent statistical significance at the 1%, 5%, and 10% levels, respectively.

As shown in column (1) of Table 5, the coefficients of the interaction terms ${\mathrm{Cohort}}_{3}^{*}$EDR were significantly negatively correlated with *EQ* at the 1% level, but the coefficients of ${\mathrm{Cohort}}_{1}^{*}$EDR, Cohort${\mathrm{Cohort}}_{2}^{*}$EDR, and ${\mathrm{Cohort}}_{4}^{*}$EDR did not have statistical significance. The estimated coefficients of Cohort^*^EDD (column 2) for each interactive variable in infancy, childhood, and adolescence are similar to those in the first column.

To compare whether the above differences in coefficients are statistically significant, we performed *t*-tests on the estimated coefficient value after regression. The statistical results are reported in the last three rows of each column in Table 5. We found that the coefficients of${\mathrm{Cohort}}_{3}^{*}$EDR were significantly bigger than those for other three interactions. This shows that the impact of CEOs' famine experience in adolescence is significantly greater than those for experience in infancy, childhood, and adulthood. In summation, the results of Table 5 indicate that CEOs' famine experience in adolescence had a significantly greater effect on corporate earnings quality, supporting H2.

## Robustness testing

Although the regression results obtained with the DID method in previous studies support the theoretical expectations of this article, the results may be distorted by endogenous problems, such as missing variables, the assumption of an equilibrium trend for the DID method, and unobservable factors at the company or macro-level. We adopted the following methods to address possible endogeneities and tested the robustness of our results.

### Common trends test

We mainly used DID to test our hypotheses. However, there is a very important prerequisite for using this method, the equilibrium trend hypothesis. Without this, the DID method cannot effectively alleviate potential endogeneity problems. Accordingly, we referred to the practices in the previous studies and designed the following model to test the equilibrium:


(4) $$\begin{array}{l} EQ_{i,t}=\beta_{0}+\beta_{1}Cohort_{-2,i,t}*EDR_{i,t}(EDD_{i,t})+\\ \;\beta_{\text{2}}Cohort_{-1,i,t}*EDR_{i,t}(EDD_{i,t})+\\ \;\beta_{\text{3}}Famine_{i,t}*EDR_{i,t}(EDD_{i,t})+Ctrls\\ \;+Y+I+P\text{+}BP+BY+\varepsilon \end{array}$$

where *Cohort*_−1_ denotes CEOs born 3 years after the famine, 1962–1964; *Cohort*_−2_ denotes CEOs born during the fourth to sixth years after the famine, 1965–1967. In model (4), we used CEOs born after 1967 as the benchmark for the equilibrium trend test. The coefficient for Cohort^*^EDR (*EDD*) represents CEOs who were born during these two periods after the famine. Assuming equilibrium trends, we expected that the coefficients of Cohort^*^EDR (*EDD*) should not be significantly different from 0.

Table 6 reports the results of regression tests using model (4). Columns (1) and (2) used Famine^*^EDR and Famine^*^EDD as variables of interest, respectively. The results in these columns indicate that the coefficients of ${\mathrm{Cohort}}_{-2}^{*}$EDR (${\mathrm{Cohort}}_{-2}^{*}$EDD) and ${\mathrm{Cohort}}_{-1}^{*}$EDR (${\mathrm{Cohort}}_{-1}^{*}$EDD) were not significant, suggesting that CEOs born after the famine have no significant differences in terms of influence on corporate earnings quality. These results are in line with the assumption about common trends in DID estimation methods. More importantly, for CEOs born before the Great Famine, the coefficients of Famine^*^EDR (Famine^*^EDR) are all significantly negative at the 5% level, indicating that CEOs' famine experience are positively associated with corporate earnings quality.

**Table 6:** CEO famine experience and corporate earnings quality tested with common trends.

	**(1)**	**(2)**
	**EQ**	**EQ**
Famine^*^EDR	−0.647^**^	
	(−2.12)	
${\mathrm{Cohort}}_{-2}^{*}$EDR	−0.300	
	(−1.36)	
${\mathrm{Cohort}}_{-1}^{*}$EDR	−0.303	
	(−1.27)	
Famine^*^EDD		−0.006^**^
		(−2.51)
${\mathrm{Cohort}}_{-2}^{*}$EDD		−0.004
		(−1.27)
${\mathrm{Cohort}}_{-1}^{*}$EDD		−0.002
		(−0.69)
Controls	Yes	Yes
Year	Yes	Yes
Industry	Yes	Yes
Province	Yes	Yes
Birth place	Yes	Yes
Birth year	Yes	Yes
*N*	23,974	23,974
Adj-R^2^	0.114	0.114

This table reports the regression results of common trends. EQ is the corporate earnings quality, calculated by the modified Jones model. Famine was encoded to 1 if the CEO was born in 1961 or earlier and 0 otherwise. Cohort_−2_ was encoded to 1 if the CEO was born 4–6 years after the famine, that is, during 1965–1967, and Cohort_−1_ was encoded to 1 if the CEO was born during 1962–1964, 3 years after the famine, respectively, and 0 otherwise. EDR is excess death ratio, valued as the mean difference of mortality in the given province during 3 years of famine subtracted from the average mortality before and after 3 years of the Great Famine. EDD is a dummy variable which encoded to 1 if the provinces have a median-above EDR during 3 years of famine and otherwise encoded to 0. Controls include Size, Lev, ROA, Grow, SOE, Audit, Dual, OCF, First, IO, Age, Gender, and Degree. Appendix 1 provides a detailed description of all the variables. The t-value is given within parentheses based on robust standard errors clustered by the firm level.

^***^, ^**^, and ^*^ represent statistical significance at the 1%, 5%, and 10% levels, respectively.

### Firm fixed effects and regional omission variables

Fixed factors of a company that do not change over time, such as honesty in the corporate culture, may also cause differences in corporate earnings quality. To control for the impact of factors that may be omitted in the regression results, we further test the main hypothesis by controlling firm fixed effects. The results are reported in the first two columns of Table 7. After controlling for these fixed effects, the coefficient for the interaction of Famine^*^EDR (Famine^*^EDD) remained significant and negative at the 5% level, which is consistent with earlier findings, indicating that the results were not sensitive to the firm-level factors that do not change over time.

**Table 7:** CEO famine experience and corporate earnings quality—the effects of firm fixed effects and regional omissions.

	**(1)**	**(2)**	**(3)**	**(4)**
	**EQ**	**EQ**	**EQ**	**EQ**
	**Firm fixed effect**		**Using neighbor province as control**	
Famine^*^EDR	−0.444^**^		−1.215^***^	
	(−2.26)		(−2.61)	
Famine^*^EDD		−0.005^**^		−0.007^**^
		(−2.45)		(−2.38)
Controls	Yes	Yes	Yes	Yes
Year	Yes	Yes	Yes	Yes
Industry	Yes	Yes	Yes	Yes
Province	Yes	Yes	Yes	Yes
Birth place	Yes	Yes	Yes	Yes
Birth year	Yes	Yes	Yes	Yes
*N*	23,974	23,974	6,612	6,612
Adj-R^2^	0.178	0.178	0.122	0.122

This table reports the regression results of firm fixed effects and regional omissions. EQ is the corporate earnings quality, calculated by the modified Jones model. Famine was encoded to 1 if the CEO was born in 1961 or earlier and 0 otherwise. EDR is the excess death ratio, valued as the mean difference of mortality in the given province during 3 years of famine subtracted from the average mortality before and after 3 years of the Great Famine. EDD is a dummy variable which is encoded to 1 if the provinces have a median-above EDR during 3 years of famine and otherwise encoded to 0. Controls include Size, Lev, ROA, Grow, SOE, Audit, Dual, OCF, First, IO, Age, Gender, and Degree. Appendix 1 provides a detailed description of all the variables. The t-value is given within parentheses based on robust standard errors clustered by the firm level.

^***^, ^**^, and ^*^ represent statistical significance at the 1%, 5%, and 10% levels, respectively.

Other factors that do not change over time may impact the conclusions of this study, such as cultural differences between different regions. To resolve this issue, we matched the provinces with higher-level *EDR* due to the famine with neighboring provinces with lower-level *EDR*. Specifically, we selected three pairs of matched samples from Shandong and Shanxi, Henan and Hebei, and Guangxi and Guangdong. These regions differ little in geography and culture, so we could use it to control some unidentified macro-level factors. The results based on the samples from the six provinces are shown in the last two columns of Table 7. After excluding the samples from other provinces, Famine^*^EDR (Famine^*^EDD) remained significantly negative at least at the 5% level, which is consistent with the previous findings.

### Propensity score matching

Differences in company characteristics, the CEO, and the region where the company is located may also cause bias. We use propensity score matching (PSM) to test the basic hypothesis. The procedures are as follows: the financial characteristics and CEO characteristics of our sample were assessed for whether the CEO experienced a famine (*Famine* = 1) and whether famine was severe in the region where the CEO was born (*EDD* = 1). A one-to-one closest-match procedure was performed to obtain two sets of highly similar samples and to eliminate differences in the earnings quality due to differences in characteristics. Subsequently, the samples were regressed. If the CEO's famine experience continues to have a positive effect on corporate earnings quality, the aforementioned indicators can be ruled out as causing differences in the corporate earnings quality.

Panel A in Table 8 reports the differences in characteristic variables between the corporate samples before and after PSM. Before PSM, the mean values of most feature variables are significantly different at the 10% level. However, after the one-to-one match, except for CEO age (*Age*), the mean differences in other characteristic variables were no longer significant, indicating that there are no differences between the treatment group and the control group. Panel B in Table 8 reports the regression results using the PSM samples. The coefficient of the CEO's famine experience variable (Famine^*^EDR and Famine^*^EDD) was still significantly negative, which is consistent with the earlier research conclusions. The matching results based on PSM showed that the promotion effect for the CEO's famine experience on the quality of corporate earnings was not due to differences in characteristic variables.

**Table 8:** Panel A: Propensity score matching parallelism hypothesis test.

	**Before PSM procedure**			**After PSM procedure**		
	***Famine* = 1**	***Famine* = 0**	**Difference**	***Famine* = 1**	***Famine* = 0**	**Difference**
Size	21.704	21.663	0.042^**^	21.702	21.692	0.010
Lev	0.485	0.472	0.014^***^	0.486	0.487	−0.001
ROA	0.030	0.032	−0.002^**^	0.030	0.029	0.001
Grow	0.125	0.158	−0.032^***^	0.126	0.125	0.001
SOE	0.656	0.505	0.152^***^	0.652	0.650	0.002
Audit	0.088	0.057	0.031^***^	0.087	0.081	0.006
Dual	0.189	0.167	0.022^***^	0.186	0.181	0.005
OCF	0.051	0.041	0.010^***^	0.050	0.048	0.002
First	0.388	0.366	0.022^***^	0.387	0.385	0.002
IO	0.159	0.187	−0.028^***^	0.160	0.160	0.000
Age	3.958	3.779	0.178^***^	3.958	3.744	0.214^***^
Gender	0.059	0.048	0.011^***^	0.057	0.054	0.003
Degree	1.424	1.454	−0.030^***^	1.433	1.435	0.002
**Panel B: CEO's famine experience and corporate earnings quality—regression results after propensity score matching**.						
	**(1)**	**(2)**
	**EQ**	**EQ**
Famine^*^EDR	−0.406^***^	
	(−3.95)	
Famine^*^EDD		−0.026^***^
		(−2.82)
Controls	Yes	Yes
Year	Yes	Yes
Industry	Yes	Yes
Province	Yes	Yes
Birth place	Yes	Yes
Birth year	Yes	Yes
*N*	9,582	9,582
Adj. R^2^	0.132	0.132

This table presents the variables descriptive statistics of PSM variables and the regression results for famine experience on earnings quality based on PSM samples. Panel A shows the variables descriptive statistics before and after PSM, and panel B shows the regress results. EQ is the corporate earnings quality, calculated by the modified Jones model. Famine was encoded to 1 if the CEO was born in 1961 or earlier and 0 otherwise. EDR is excess death ratio, valued as the mean difference of mortality in the given province during 3 years of famine subtracted from the average mortality before and after 3 years of the Great Famine. EDD is a dummy variable which is encoded to 1 if the provinces have a median-above EDR during 3 years of famine and otherwise encoded to 0. Controls include Size, Lev, ROA, Grow, SOE, Audit, Dual, OCF, First, IO, Age, Gender, and Degree. Appendix 1 provides a detailed description of all the variables. The t-value is given within parentheses based on robust standard errors clustered by the firm level.

^***^, ^**^, and ^*^ represent statistical significance at the 1%, 5%, and 10% levels, respectively.

### Other robustness tests

#### CEOs' distribution

As given in Table 2, the number of samples from each region is different, and too many and too few CEO samples from the same region may also affect our regression results. Hence, we deleted the three largest and three smallest provinces and performed a regression on the remaining. The results are given in Table 9. Columns (1) and (2) exclude Guangdong, Jiangsu, and Zhejiang, the three provinces with the largest numbers of CEO births. Columns (3) and (4) exclude Qinghai, Ningxia, and Hainan, the three provinces with smallest numbers of CEO births. Columns (5) and (6) exclude both the sets. In these estimates, Famine^*^EDR (Famine^*^EDD) remained significantly negative at least at the 1% level, which is consistent with the previous conclusions, indicating that the impact of the famine experience on the quality of corporate earnings was not due to the region of birth.

**Table 9:** CEOs' famine experience and the quality of corporate earnings—the impact of excluding CEO samples.

	**(1)**	**(2)**	**(3)**	**(4)**	**(5)**	**(6)**
	**EQ**	**EQ**	**EQ**	**EQ**	**EQ**	**EQ**
	**Delete upper three provinces**		**Delete bottom three provinces**		**Delete both sets**	
Famine^*^EDR	−0.296^**^		−0.271^**^		−0.290^**^	
	(−2.05)		(−1.98)		(−1.99)	
Famine^*^EDD		−0.006^***^		−0.006^***^		−0.006^***^
		(−3.63)		(−3.62)		(−3.58)
Controls	Yes	Yes	Yes	Yes	Yes	Yes
Year	Yes	Yes	Yes	Yes	Yes	Yes
Industry	Yes	Yes	Yes	Yes	Yes	Yes
Province	Yes	Yes	Yes	Yes	Yes	Yes
Birth place	Yes	Yes	Yes	Yes	Yes	Yes
Birth year	Yes	Yes	Yes	Yes	Yes	Yes
*N*	17,795	17,795	23,610	23,610	17,431	17,431
Adj-R^2^	0.115	0.115	0.114	0.114	0.115	0.116

This table reports the regression results of excluding some special CEO samples. EQ is the corporate earnings quality, calculated by the modified Jones model. Famine was encoded to 1 if the CEO was born in 1961 or earlier and 0 otherwise. EDR is excess death ratio, valued as the mean difference of mortality in the given province during 3 years or famine subtracted from the average mortality before and after 3 years of the Great Famine. EDD is a dummy variable which is encoded to 1 if the provinces have a median-above EDR during 3 years of famine and otherwise encoded to 0. Controls include Size, Lev, ROA, Grow, SOE, Audit, Dual, OCF, First, IO, Age, Gender, and Degree. Appendix 1 provides a detailed description of all the variables. The t-value is given within parentheses based on robust standard errors clustered by the firm level.

^***^, ^**^, and ^*^ represent statistical significance at the 1%, 5%, and 10% levels, respectively.

#### Sensitivity of earnings quality indicators

In the previous test, we used the modified Jones model as a proxy for earnings quality. To test whether the results are sensitive to the measurements of earnings quality, we retested the basic assumptions with different measures. First, the method of Dechow and Dichev (2002) was used to consider the impact of current, past, and future cash flows to measure earnings quality. Second, following Kothari et al. (2005), we used the Jones model modified by performance to measure earnings quality. Third, referring to McNichols (2002), we used a comprehensive DD model (Dechow and Dichev, 2002) and a modified Jones model to measure earnings quality.

Table 10 reports the regression results for these three indicators to measure corporate earnings quality. For all six columns, the coefficients of the interaction term Famine^*^EDR (Famine^*^EDD) are all significantly negative at the 5% level, which is consistent with the previous results, indicating that CEOs' famine experience improves corporate earnings quality. The results given in Table 10 also indicate that changing the measurement of the earnings quality did not affect our basic conclusions.

**Table 10:** CEOs' famine experience and the quality of corporate earnings—sensitivity test for the proxies of earnings quality.

	**(1)**	**(2)**	**(3)**	**(4)**	**(5)**	**(6)**
	**EQ_1_**	**EQ_1_**	**EQ_2_**	**EQ_2_**	**EQ_3_**	**EQ_3_**
	**DD model**		**ROA adjust Jones Model**		**DD and Jones Model**	
Famine^*^EDR	−0.294^**^		−0.306^**^		−0.373^***^	
	(−2.21)		(−2.15)		(−2.63)	
Famine^*^EDD		−0.006^***^		−0.005^**^		−0.005^***^
		(−3.84)		(−2.47)		(−2.58)
Controls	Yes	Yes	Yes	Yes	Yes	Yes
Year	Yes	Yes	Yes	Yes	Yes	Yes
Industry	Yes	Yes	Yes	Yes	Yes	Yes
Province	Yes	Yes	Yes	Yes	Yes	Yes
Birth place	Yes	Yes	Yes	Yes	Yes	Yes
Birth year	Yes	Yes	Yes	Yes	Yes	Yes
*N*	23,974	23,974	22,473	22,473	22,473	22,473
Adj-R^2^	0.114	0.114	0.191	0.191	0.123	0.123

This table reports the regression results of the sensitivity test for the proxies of earnings quality. EQ is the corporate earnings quality, which is calculated by the DD model in the first two columns, ROA adjust Jones model in the middle two columns, and DD and Jones model in the last two columns. Famine was encoded to 1 if the CEO was born in 1961 or earlier and 0 otherwise. EDR is the excess death ratio, valued as the mean difference of mortality in the given province during 3 years of famine subtracted from the average mortality before and after 3 years of the Great Famine. EDD is a dummy variable which is encoded to 1 if the provinces have a median-above EDR during 3 years of famine and otherwise encoded to 0. Controls include Size, Lev, ROA, Grow, SOE, Audit, Dual, OCF, First, IO, Age, Gender, and Degree. Appendix 1 provides a detailed description of all the variables. The t-value is given within parentheses based on robust standard errors clustered by the firm level.

^***^, ^**^, and ^*^ represent statistical significance at the 1%, 5%, and 10% levels, respectively.

#### Considering impact of the new accounting standards and some omitted factors

The period of our sample begins in 2000, which may ignore the huge potential impact of the new accounting standards for enterprises in 2007 on earnings quality. In our previous model, we have controlled the annual fixed effect to control these factors. We conducted a new robustness test using the sub-sample after 2007 in this part. The results are shown in columns (1) and (2) of Table 11. We found that the coefficients of *Famine* ^*^ *EDR* and *Famine* ^*^ *EDD* are −0.632 and −0.010, respectively. They were significant at the 1% level, which still supports our hypothesis.

**Table 11:** CEOs' famine experience and the quality of corporate earnings—using sub-sample after 2007 and adding more fixed effects.

	**(1)**	**(2)**	**(3)**	**(4)**
	**EQ**	**EQ**	**EQ**	**EQ**
	**Using samples after 2007**		**Control more fixed effect**	
Famine^*^EDR	−0.632^***^		−0.315^**^	
	(−3.32)		(−2.35)	
Famine^*^EDD		−0.010^***^		−0.006^***^
		(−4.85)		(−4.22)
Year	Yes	Yes	Yes	Yes
Industry	Yes	Yes	Yes	Yes
Province	Yes	Yes	Yes	Yes
Year^*^Industry	Yes	Yes	Yes	Yes
Year^*^Province	Yes	Yes	Yes	Yes
Birth place	Yes	Yes	Yes	Yes
Birth year	Yes	Yes	Yes	Yes
*N*	15,310	15,310	23,974	23,974
Adj-R^2^	0.145	0.146	0.125	0.126

This table reports the regression results of the sensitivity test using the sub-sample after 2007 and adding more fixed effects. EQ is the corporate earnings quality, calculated by the modified Jones model. Famine was encoded to 1 if the CEO was born in 1961 or earlier and 0 otherwise. EDR is excess death ratio, valued as the mean difference of mortality in the given province during 3 years of famine subtracted from the average mortality before and after 3 years of the Great Famine. EDD is a dummy variable that is encoded to 1 if the provinces have a median-above EDR during 3 years of famine and otherwise encoded to 0. Controls include Size, Lev, ROA, Grow, SOE, Audit, Dual, OCF, First, IO, Age, Gender, and Degree. Appendix 1 provides a detailed description of all the variables. The t-value is given within parentheses based on robust standard errors clustered by the firm level.

^***^, ^**^, and ^*^ represent statistical significance at the 1%, 5%, and 10% levels, respectively.

Addition, some factors that have a strong influence on the motivation of earnings management are not included in the model, such as the motivation to avoid losses. We added more fixed effects to the model to weaken the possible impact of these missing variables. In columns (3) and (4), we conducted a robustness test to control the fixed effects of *Year* ^*^ *Industry* and *Year* ^*^ *Province*. The results show that the coefficients of *Famine* ^*^ *EDR* and *Famine* ^*^ *EDD* are significantly negative at least at the 5% level. The results given in Table 11 also indicate that our conclusions are relatively robust.

## Further analyses

The aforementioned regression results showed that CEOs' famine experience was significantly positively associated with corporate earnings quality. As detailed in hypothesis 1, we considered two potential mechanisms: the risk prevention effect and the famine learning effect. Next, to reveal the internal mechanism of CEOs' famine experience affecting the corporate earnings quality, we further tested the potential mechanisms.

### Risk prevention effects

Risk prevention effects assume that the early-life famine experience of CEOs makes them more risk-sensitive and risk-averse. Low-quality earnings information disclosure may lead to a higher risk of litigation. Therefore, CEO's manipulation of earnings information is more cautious, and the quality of corresponding earnings information disclosure is thus relatively high. Based on the above analysis, if the CEO's early famine experience affected the corporate earnings quality mainly through risk prevention effects, it can be expected that when a company's low-quality earnings information disclosure incurs relatively higher risks, the CEO's risk prevention motivation would be relatively stronger, and the association between the CEO's famine experience and corporate earnings quality should be more obvious.

We measured risk intensity in terms of investor protection and whether the company was cross-listed. Regarding investor protection, the existing literature shows that in regions with high levels of investor protection, laws and regulations are more easily met, so the degree of coordination between laws and market mechanisms is relatively high, and investors' awareness of protections is more visible and intense (Klapper and Love, 2004). In this context, the potential risk intensity for low-quality information disclosure is higher. For cross-listed companies, the regulatory environment of cross-listed companies is more stringent, and the awareness of investor protections is also higher (Ke et al., 2015). This means that cross-listed companies face more risk in relation to low-quality earnings information disclosure.

Based on the above considerations, we used the investor protection index (IPI) from the China Marketization Index Report prepared by Fan et al. (2011) to measure the degree of investor protection. Meanwhile, we used whether the company is cross-listed in both A and H shares (AH) to measure cross-listing. We divided our sample into two groups to test the risk prevention effects of CEOs' experience of the Great Famine. Table 12 reports the regression results. In the first four columns, the coefficients of Famine^*^EDR and Famine^*^EDD are negatively associated with *EQ* in two low-IPI groups (where IPI is less than the value of the median), while in the high-IPI group, the Famine^*^EDR is not significant but Famine^*^EDD is significant at the 10% level. In the last four columns, the results show that Famine^*^EDR and Famine^*^EDD are all negative at least at the 10% level in the AH list group and only A list group. We further tested the coefficient differences between these divided groups using the chi-square method. The results indicated that the absolute value of the coefficient of Famine^*^EDR and Famine^*^EDD in the high-IPI (AH list) group is significantly larger than that for the low-IPI (A list only) group. These results suggested that when the risk intensity for low-quality information disclosure is higher, CEOs' famine experience has a more significant effect on the corporate earnings quality, which is consistent with our expectations regarding the risk prevention effect.

**Table 12:** CEO famine experience and quality of corporate earnings—a test of risk aversion effects.

	**(1)**	**(2)**	**(3)**	**(4)**	**(5)**	**(6)**	**(7)**	**(8)**
	**EQ**	**EQ**	**EQ**	**EQ**	**EQ**	**EQ**	**EQ**	**EQ**
	**Low IPI**	**High IPI**	**Low IPI**	**High IPI**	**A list only**	**AH list**	**A list only**	**AH list**
Famine^*^EDR	−0.007	−0.711^***^			−0.134^*^	−0.979^**^		
	(−0.04)	(−3.22)			(−1.66)	(−2.46)		
Famine^*^ EDD			−0.004^*^	−0.010^***^			−0.004^**^	−0.014^***^
			(−1.90)	(−4.33)			(−2.46)	(−2.62)
*Differences*	Chi^2^ = 6.95^***^		Chi^2^ = 4.43^**^		Chi^2^ = 3.11^*^		Chi^2^ = 3.17^*^	
	(*P* > Chi^2^ = 0.008)		(*P* > Chi^2^ = 0.036)		(*P* > Chi^2^ = 0.078)		(*P* > Chi^2^ = 0.075)	
Controls	Yes	Yes	Yes	Yes	Yes	Yes	Yes	Yes
Year	Yes	Yes	Yes	Yes	Yes	Yes	Yes	Yes
Industry	Yes	Yes	Yes	Yes	Yes	Yes	Yes	Yes
Province	Yes	Yes	Yes	Yes	Yes	Yes	Yes	Yes
Birth place	Yes	Yes	Yes	Yes	Yes	Yes	Yes	Yes
Birth year	Yes	Yes	Yes	Yes	Yes	Yes	Yes	Yes
*N*	11,787	12,187	11,787	12,187	22,930	1,044	22,930	1,044
Adj-R^2^	0.109	0.127	0.109	0.127	0.113	0.238	0.113	0.242

This table reports the regression results of risk aversion effects. EQ is the corporate earnings quality, calculated by the modified Jones model. Famine was encoded to 1 if the CEO was born in 1961 or earlier, and 0 otherwise. EDR is excess death ratio, valued as the mean difference of mortality in the given province during 3 years of famine subtracted from the average mortality before and after 3 years of the Great Famine. EDD is a dummy variable which is encoded to 1 if the provinces have a median-above EDR during 3 years of famine and otherwise encoded to 0. IPI measures the degree of investor protection index prepared by Fan et al. (2011), high IPI means the province in 1 year got a median-above IPI compared to others. A List only means firms only listed A share market in China, while AH List means cross-list with A and H share. Controls include Size, Lev, ROA, Grow, SOE, Audit, Dual, OCF, First, IO, Age, Gender, and Degree. Appendix 1 provides a detailed description of all the variables. The t-value is given within parentheses based on robust standard errors clustered by the firm level.

^***^, ^**^, and ^*^ represent statistical significance at the 1%, 5%, and 10% levels, respectively.

### Famine learning effect

The famine learning effect holds that the epochal characteristics and reasons behind the occurrence of the Great Famine increase the knowledge of famine-experienced CEOs. Under the influence of the learning effect, the probability of the CEO to implement earnings manipulation and disclose untrue earnings information is lower, resulting in higher quality earnings information disclosure. Based on the above analysis, if CEOs' early famine experience affected the quality of corporate earnings, mainly through the famine learning effect, it can be expected that when the corporate CEOs' learning ability and knowledge accumulation of the famine event are relatively higher, the association between CEOs' famine experience and corporate earnings quality should be more obvious.

We used CEO education and professional background to measure CEOs' learning ability and knowledge accumulation related to the early famine event. Jiang et al. (2016) found that when the CEO has more education, the secretary's financial experience plays a larger role in information disclosure, and earnings information of the company is of higher quality. Following their research, we expected that when the CEO's education is higher, the learning effect of the famine event should be more obvious. When the professional background of the CEO is in economics or management, the CEO can better understand the reasons for the occurrence of the Great Famine and could learn more from the disaster.

Based on CEO education and professional background data compiled in the CSMAR database, we manually collected samples to supplement the missing data as much as possible. To measure CEO education (*Degree*), we followed Jiang et al. (2016) again and divided academic education into five levels: high school, junior college, undergraduate degree, masters, and doctorate levels; then, we defined CEOs with a master's or doctorate degree as the high-degree group, and the remaining as the low-degree group. Meanwhile, when the CEOs' major was in economics or management, we assigned these CEOs to the E&M group; otherwise, they were assigned to the non-E&M group. Table 13 reports the results of the CEOs' famine learning effect. In the first four columns, the coefficient of the interaction term Famine^*^EDR (Famine^*^EDD) is −0.832 (−0.104) in the high-degree group and is significant at the 1% level, while in the low-degree group, the coefficient is not significant. In the remaining four columns, the coefficients of Famine^*^EDR and Famine^*^EDD in the E&M group are negatively significant at the 5 and 1% levels, respectively, while in the non-E&M group, the Famine^*^EDR is not significant, but Famine^*^EDD is significant at the 5% level. Using the chi-square method, we further test the differences of coefficients between these two groups. We found that the absolute value of the coefficient for Famine^*^EDR and Famine^*^EDD in the high-degree (E&M) group is significantly larger than that for the low-degree (non-E&M) group. In summation, these results indicated that when the CEO's learning ability and knowledge accumulation are relatively higher, the famine experience has a more significant effect on corporate earnings quality, which hold against the expectations of the famine learning effect.

**Table 13:** CEO famine experience and the quality of corporate earnings—a test of famine learning effects.

	**(1)**	**(2)**	**(3)**	**(4)**	**(5)**	**(6)**	**(7)**	**(8)**
	**EQ**	**EQ**	**EQ**	**EQ**	**EQ**	**EQ**	**EQ**	**EQ**
	**Low degree**	**High degree**	**Low degree**	**High degree**	**Non EandM**	**EandM**	**Non EandM**	**EandM**
Famine^*^EDR	−0.008	−0.832^***^			−0.147	−1.037^**^		
	(−0.03)	(−3.60)			(−0.85)	(−3.16)		
Famine^*^ EDD			−0.001	−0.014^***^			−0.004^**^	−0.017^***^
			(−0.73)	(−5.48)			(−2.38)	(−4.79)
*Differences*	Chi^2^ = 8.66^***^		Chi^2^ = 15.55^***^		Chi^2^ = 6.25^**^		Chi^2^ = 11.46^***^	
	(*P* > Chi^2^ = 0.003)		(*P* > Chi^2^ < 0.001)		(*P* >Chi^2^ = 0.012)		(*P* > Chi^2^ < 0.001)	
Controls	Yes	Yes	Yes	Yes	Yes	Yes	Yes	Yes
Year	Yes	Yes	Yes	Yes	Yes	Yes	Yes	Yes
Industry	Yes	Yes	Yes	Yes	Yes	Yes	Yes	Yes
Province	Yes	Yes	Yes	Yes	Yes	Yes	Yes	Yes
Birthplace	Yes	Yes	Yes	Yes	Yes	Yes	Yes	Yes
Birth year	Yes	Yes	Yes	Yes	Yes	Yes	Yes	Yes
*N*	16,066	7,908	16,066	7,908	9,173	4,439	9,173	4,439
Adj-R^2^	0.111	0.131	0.111	0.134	0.121	0.238	0.122	0.204

This table reports the regression results of famine learning effects. EQ is the corporate earnings quality, calculated by the modified Jones model. Famine was encoded to 1 if the CEO was born in 1961 or earlier and 0 otherwise. EDR is excess death ratio, valued as the mean difference of mortality in the given province during 3 years of famine subtracted from the average mortality before and after 3 years of the Great Famine. EDD is a dummy variable which is encoded to 1 if the provinces have a median-above EDR during 3 years of famine and otherwise encoded to 0. Degree means the degree of CEOs. High Degree means the CEOs got a master's and doctorate degrees. EandM means the CEOs' major was in economics or management and otherwise assigned to non-EandM group. Controls include Size, Lev, ROA, Grow, SOE, Audit, Dual, OCF, First, IO, Age, Gender, and Degree. Appendix 1 provides a detailed description of all the variables. The t-value is given within parentheses based on robust standard errors clustered by the firm level.

^***^, ^**^, and ^*^ represent statistical significance at the 1%, 5%, and 10% levels, respectively.

## Discussion

### Main findings

Using the exogenous event of the Great Chinese Famine as the starting point, this research examined the impact of CEOs' experience of this famine on corporate earnings quality. This research found that CEOs who experienced this event led to higher earnings quality for their companies. We also showed that this effect is more pronounced for CEOs who experienced this famine during their childhood or adolescence. On the one hand, CEOs who experienced the famine showed a higher risk aversion behavior. When the degree of investor protection is high and the company is cross-listed, the positive effects of the famine experience on earnings quality are more obvious. On the other hand, CEOs show a certain learning effect related to the disaster. More education or professional background in economics or management led to significantly better corporate earnings quality for companies with famine-experienced CEOs. The above findings prove that the CEOs' famine experiences affect the earnings quality through the risk aversion effect and famine learning effect. These conclusions were validated through a series of robustness tests.

### Theoretical implications

The results of the current study are consistent with those of previous research, and this study makes several contributions to the literature. First, taking CEOs' heterogeneity into account, our paper provides empirical evidence that CEOs' early-life famine experience is significantly positively associated with corporate earnings quality, thereby further enriching and supplementing the relevant areas of the literature on corporate earnings quality. Second, due to the exogenous and unpredictable nature of the Great Famine, our paper also provides a more robust estimate of the impact of CEOs' early-life experiences on corporate decision making, thereby addressing the selection bias in the existing literature that considered financial experience, overseas experience, and military experience of CEOs. Third, we explored the mechanism of CEOs' famine experiences affecting the earnings quality, and we found that the risk aversion effect and famine learning effect are the two mechanisms of CEOs' famine experiences affecting earnings quality. These findings enrich the literature on the long-term economic consequences of the Great Chinese Famine.

### Practical implications

We can draw some practical implications from our research results. The impact of informal institutions on corporate governance is attracting increased attention. Our paper indicated that CEOs' famine experience operates in a similar manner as an implicit contract, according to which managers seek to produce high-quality outputs in their decision-making process. When an enterprise operates in an environment of high uncertainty, this implicit contract improves governance.

## Limitation and future research

This study has several limitations. First, the family background is an important factor influencing CEO's perception of the Great Chinese Famine. If the manager came from a relatively well-off family in comparison to the average financial situation at the time, the famine experience may have little impact on them. On the contrary, CEOs who came from a poor family may have a deep impression on the Great Chinese Famine. However, it is difficult for us to obtain the CEO's family data, such as income and their parents' work. Second, it is not just only a CEO who can decide corporate's behavior, and the corporate's behavior is also influenced by other factors, such as the board of directors. In practice, other managers and independent directors also can affect the corporate's economic decision making. Therefore, it may not be enough to just take CEO as the research object. If we conduct research based on the management team, it is difficult to construct the indicators of the management team's famine experience at the team level.

In future research, it is expected that more information about CEOs and their family background can be obtained through big data or field research. It is also expected to explore some new famine experience indicators at the management team level. We are looking forward to providing more causal evidence on the impact of famine experience on corporate behavior.

## Data availability statement

The raw data supporting the conclusions of this article will be made available by the authors, without undue reservation.

## Author contributions

YZ contributed significantly to analysis and manuscript preparation. JH performed the data analyses and wrote the manuscript. LL helped perform the analysis with constructive discussions. All authors contributed to the conception of the study and approved the submitted version.

## Funding

We acknowledge the support from the National Nature Science Foundation of China (Grant Number: 72102149) and the 2021 project of Guangzhou Planning of Philosophy and Social Science (Grant Number: 2021GZQN05).

## Conflict of interest

The authors declare that the research was conducted in the absence of any commercial or financial relationships that could be construed as a potential conflict of interest.

## Publisher's note

All claims expressed in this article are solely those of the authors and do not necessarily represent those of their affiliated organizations, or those of the publisher, the editors and the reviewers. Any product that may be evaluated in this article, or claim that may be made by its manufacturer, is not guaranteed or endorsed by the publisher.

## Appendix 1

**Table:** Definitions and methods of calculation for the main variables.

**Symbol**	**Name**	**Definition and method of calculation**
EQ	Earnings quality	Modified Jones model; the method of calculation is described in the introduction
Famine	Famine experience	1 if the CEO was born in 1961 or earlier, 0 otherwise
Cohort_1_	Infancy	1 if the CEO was born between 1959 and 1961, 0 otherwise
Cohort_2_	Childhood	1 if the CEO was born between 1955 and 1958, 0 otherwise
Cohort_3_	Teenager	1 if the CEO was born between 1944 and 1954, 0 otherwise
Cohort_4_	Adult	1 if the CEO was born before 1944, 0 otherwise
EDR	Excess mortality	Excess mortality, valued as the difference in mortality in the given province for the 3 years of the famine and the average for the previous 3 years overall
EDD	Excess mortality	1 if EDR_1_ is higher than the median, 0 otherwise
Size	Company size	Natural logarithm of company's total assets at the end of the year
Lev	Financial leverage	Gearing ratio, the company's total liabilities at the end of the year divided by total assets at the end of the year
Roa	Profitability	Return on assets, calculated as net profit divided by total assets at the end of the year
Grow	Company growth	Sales revenue growth rate, the annual sales revenue minus the previous year's sales revenue divided by the previous year's sales revenue
Soe	Property right	1 if the company is a state-owned enterprise and 0 otherwise
Big	Big Four	1 if the corporate auditor is in the Big Four, otherwise Big is assigned a value of 0
Dual	Two jobs in one	1 for CEOs who are also chairs and 0 otherwise
Ocf	Free cash flow	Net cash flow generated by the company at the end of the year divided by total assets at the end of the period
First	Major shareholder holdings	Number of shares held by the company's largest shareholders divided by total number of shares
Inst	corporate investor	Number of shares held by institutional investors divided by total shares
Age	CEO age	Natural logarithm of CEO age
Gender	CEO gender	1 if CEO is female and 0 if male
Degree	CEO degree	Natural logarithm of population in the province where the business is located
